# A Case of Acute Viral Pericarditis Complicated With Pericardial Effusion Induced by Third Dose of COVID Vaccination

**DOI:** 10.7759/cureus.21207

**Published:** 2022-01-13

**Authors:** Hany A Zaki, Adel Zahran, Mohammed Abdelrahim, Wael Abdelrehem Elnabawy, Yasser Kaber

**Affiliations:** 1 Emergency Medicine, Hamad Medical Corporation, DOHA, QAT; 2 Emergency Medicine, Hamad Medical Corporation, Doha, QAT; 3 Emergency Department, Hamad Medical Corporation, Doha, QAT

**Keywords:** sars-cov-2, mrna vaccines, covid-19 vaccination, pericardial effusion, viral pericarditis

## Abstract

COVID-19 vaccines were safe and efficacious in clinical trials. A two-dose regimen of the Pfizer-BioNTech COVID-19 vaccine confers no less than 95% protection against COVID-19 with an adequate safety profile. To date, no reports have been made in the literature regarding the onset of acute viral pericarditis after vaccination with the Pfizer BNT162b2 vaccine. But on the other hand, pericarditis is reported to occur in rare instances of COVID-19 infection, and this may be attributed to the pro-inflammatory effects of the spike protein. In this article, we describe the case of an elderly male patient with a known case of hypothyroidism who presented to our emergency department with fever, chills, and dry cough for ten days after the third dose of the Pfizer-BioNTech COVID-19 vaccine. Although we cannot mention a direct effect, it is essential to note a potential adverse reaction to vaccine administration following the expression of SARS-CoV-2 spike protein-induced from the vaccine’s mRNA.

## Introduction

Acute viral pericarditis is the commonest form of inflammatory cardiac disease, with an estimated yearly incidence of 28 cases per 100,000 subjects in Western nations [1]. The most accepted etiology is a presumed or definite viral infection. It is important to note that severe acute respiratory syndrome (SARS-CoV-2) was a novel etiologic agent of varying pericardial symptoms, such as acute pericarditis.

In the absence of an effective therapeutic agent against the disease, mass vaccination remains the most exclusive means of controlling the pandemic. It is worth mentioning that several vaccines, all highly effective and designed to prevent COVID-19-related deaths and hospitalizations, were mass-produced and distributed in record time. Several rare complications due to vaccine administration have been reported.

The Pfizer-BioNTech COVID-19 mRNA vaccine, for instance, is an mRNA-based vaccine that protects against infection by SARS-CoV-2 in people [2]. The vaccine is based on spike RBA expression, which expresses the protein after inoculation and stimulates its recognition by immune cells. It is a known fact that a two-dose regimen of the BNT162b2 vaccine confers at least 95% protection against COVID-19 with a good safety profile [3]. The commonest adverse reactions reported in the literature include fatigue, pain at the injection site, headache, chills, myalgia, Bell’s paralysis, arthralgia, and fever [4].

This case report describes acute pericarditis as a potential complication of COVID-19 vaccination, focusing on the interval between the vaccination and onset of symptoms, clinical manifestations, short-term outcomes, and peculiar features.

## Case presentation

A 55-year-old male patient known only to have hypothyroidism was presented to our emergency department with chills, fever, and dry cough for ten days, after receiving his third dose of COVID-19 vaccine ( Pfizer-BioNTechCOVID-19 mRNA) by two days. Vital signs as observed included: Temperature 37.7°, Oxygen saturation - 97% room air, Blood pressure - 140/95 mmHg, Heart rate - 88 beats per minute, Respiratory rate - 14 breaths per minute

**Figure 1:**
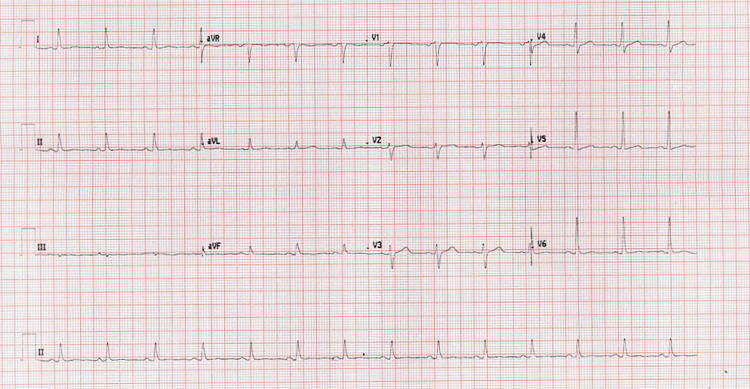
Electrocardiography (ECG) showed a regular sinus rhythm with an 80 beats/minute ventricular rate, with no evidence of ischemia or arrhythmia.

Physical examination showed that the patient appears well, apyrexial, and without jaundice/anemia/cyanosis/clubbing/lymphadenopathy, maintaining saturation on room air, and not in pain or distress. Examination of the cardiovascular system showed a regular radial pulse, normal character, blood pressure as per vital signs, JVP not raised, and heart sounds first and second with friction rub. ECG has been done with standard variant (figure 1).

**Figure 2:**
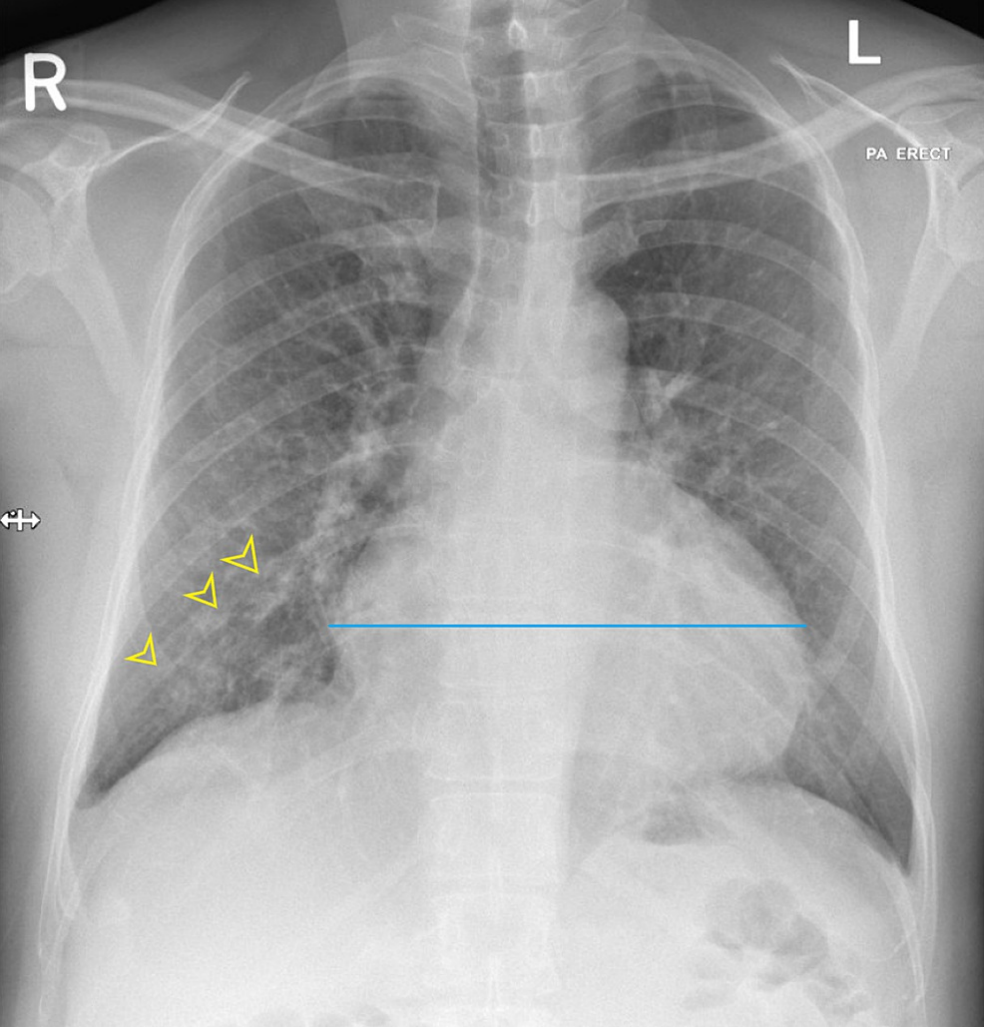
Chest X-ray (PA and erect view) showed cardiac size is enlarged with biventricular configuration (The blue line), also evidence of infiltration on the right lower lung zone (yellow arrowheads) suggestive of pneumonia.

Examination of the respiratory system showed no increased breathing work, and percussion notes resonant, chest sounds were equal and bilateral. Examination of the gastrointestinal, genitourinary & abdominal systems was soft and non-tender. Central nervous system examination showed that the patient appears alert, Glasgow Coma Scale/Score (GCS) 15/15, no focal neurology. CXR was done at the health center and showed pneumonia (figure 2).

The patient’s CURB-65 score (Confusion, Urea nitrogen, Respiratory rate, Blood pressure, 65 years of age and older) was zero, while blood investigations were unremarkable. A decision for discharge with symptomatic treatment was taken.

The next day, the patient presented again to the emergency facility with worsening dry cough and high-grade fever 39.9°. There was pain on the left side of the chest, and the pain increased when the patient lay flat and was relieved with leaning forward. The patient denied shortness of breath. There was no palpitation, syncope, or lower limb edema-the patient reported a family history of a similar condition with a daughter diagnosed with pericardial effusion.

**Figure 3:**
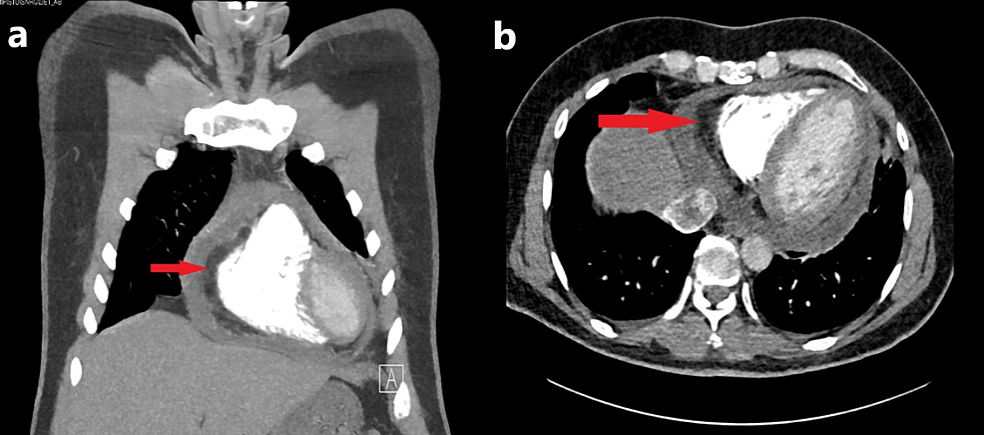
Computed tomography pulmonary angiography (a) Coronal view showed clear evidence of moderate pericardial effusion (red arrows), and (b) axial view of the chest showed also clear evidence of moderate pericardial effusion (red arrow).

Examination showed normal sinus rhythm. Blood investigations were repeated and showed moderate microcytic hypochromic anemia, D-dimer 19.74, C-Reactive Protein (CRP) test 180.9. CT pulmonary angiography was conducted (figure 3) with the following conclusion: No CT evidence of pulmonary edema, Bilateral ill-defined ground-glass infiltrates, Moderate pericardial effusion.

**Figure 4:**
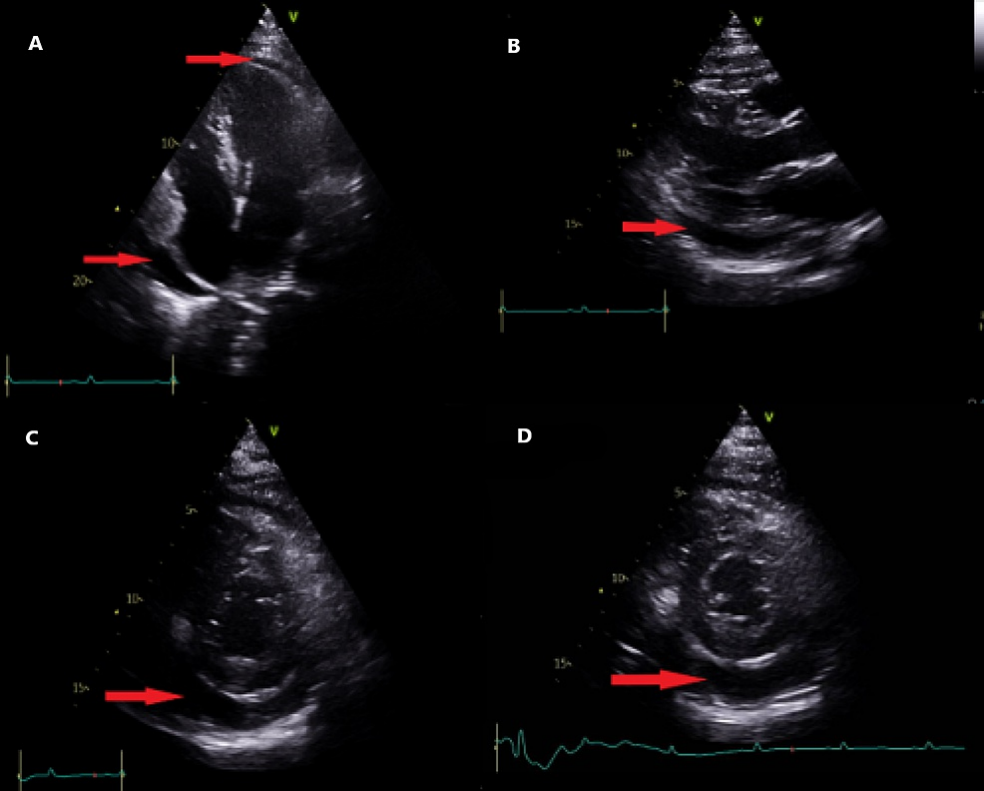
Transthoracic cardiac echocardiography four chambers view (a), parasternal long-axis view (b), and parasternal short-axis view (c) and (d) all showed clear evidence of pericardial effusion (red arrows).

Transthoracic echocardiography (figure 4) was done and showed the great vessel: IVC poorly visualized. Pericardium: mild circumferential pericardial effusion. For clinical correlation, pericardial effusion is more marked near the right atrium-no echocardiographic evidence of tamponade. Respiratory variation was greater than 40% of Tidal volume (TV) inflow. Follow-up Transthoracic echocardiography (TTE) was recommended.

Later, the medical register was consulted for admission, and the patient was admitted as a case of chest pain with pericardial effusion, mostly viral pericarditis. The patient was reviewed by the cardiologist with the following management plan: To start Colchicine 0.5mg orally if no contraindication, Rabeprazole 20mg orally once daily, Ibuprofen 600mg orally BID if no contraindication, Start IV ceftriaxone and azithromycin and follow sepsis work-up result, VTE prophylaxis: enoxaparin. 

The patient was discharged after a clinically improved hospital stay for a few days with referral to cardiology clinic as a case of viral pericarditis post-covid vaccination.

## Discussion

What makes this case peculiar is that a healthy male who received a vaccine against COVID-19 (with the Pfizer-BioNTech COVID-19 mRNA vaccine) developed signs and symptoms of acute pericarditis ~ 10 days after vaccination. After meeting some diagnostic criteria, the patient was diagnosed with acute pericarditis, namely pericardial perfusion and typical pain [5].

As indicated in most recent guidelines [5,6], colchicine and anti-inflammatory therapy proved effective and safe in reducing symptoms. The patient had a favorable clinical course without complications such as tamponade, pericardial effusion, myopericarditis, high inflammatory indices, and a positive response to treatment with colchicine [7]. Transthoracic echocardiography confirmed the regression of pericarditis.

Pericarditis following COVID-19 vaccinations appears to be more prevalent among men for AD26.COV2. S. and mRNA vaccines. It is important to note that most patients are recovering/recovering regarding the outcome. Some experience a complicated disease course, while some fatalities are recorded following vaccination with mRNA vaccines [8]. So, although it may be rare, the association of pericarditis with COVID-19 vaccination should be considered a serious one.

It is worth mentioning here that Ad26.COV2. S and AZD1222 comprise a recombinant replication-incompetent human adenovirus vector (type 26) or simian (chimpanzee) vector, respectively. Both encode the SARS-CoV-2 spike protein [9,10]. Adenoviruses infect the pericardium and trigger pericarditis. Regarding vaccination with ad-vectored vaccines, the backbones (adenoviruses) are unable to replicate, but hypothetically can infect the pericardium, causing extremely high expression of the spike protein, since there are over 5 x 1010 viral particles in each vaccine; this may result in acute toxicity via innate immune response activation, as described in the literature [9-11].

Extra RNA species may also trigger innate immune responses leading to inflammatory reactions. These RNA species are contained in mRNA vaccines and stem from various factors, including initial manufacturing stages, suboptimal late manufacturing stage, or storage conditions, alongside the inherent instability which the RNA molecule is known for [12].

Single and double-stranded RNA may present as by-products during in vitro transcription and may be identified by innate immune sensors, like cytoplasmic protein kinases and toll-like receptors [12]. Modification of the nucleotide composition of the RNA to contain N 1-methylpseudouridine rather than uridine reduces but does not eliminate undesired responses from the immune system. It is also important to mention that despite mRNA encapsulation in lipid nanoparticles (LNP), the mRNA-LNP moiety can degrade, mostly under ultra-frozen conditions (typically non-recommended), or during thawing of the vaccine batches for administration.

Early batches of vaccines that got first approval (BNT162b2) were discovered to contain very low mRNA levels (12). Manufacturers should adhere meticulously to good manufacturing practices, especially when the mRNA is purified from in vitro contaminants and for quality control assurance of every dose. There is also a need for improvements in the mRNA-LNP stability of the product in order to prevent these rare complications associated with the administration of the vaccine.

## Conclusions

The involvement of the cardiac system without pulmonary findings may be something to consider in symptomatic individuals who have received the COVID-19 vaccine. Acute pericarditis is typically underdiagnosed. The onset of acute pericarditis due to COVID-19 vaccination may be caused by an underlying immune-mediated factor.

## References

[1] Adler Y, Charron P, Imazio M (2015). 2015 ESC Guidelines for the diagnosis and management of pericardial diseases: The task force for the diagnosis and management of pericardial diseases of the European Society of Cardiology (ESC) endorsed by: The European Association for Cardio-Thoracic Surgery (EACTS). Eur Heart J.

[2] Rosenblum HG, Hadler SC, Moulia D (2021). Use of COVID-19 vaccines after reports of adverse events among adult recipients of Janssen (Johnson & Johnson) and mRNA COVID-19 Vaccines (Pfizer-BioNTech and Moderna): update from the advisory committee on immunization practices - United States, July 2021. MMWR Morb Mortal Wkly Rep.

[3] Polack FP, Thomas SJ, Kitchin N (2020). Safety and efficacy of the BNT162b2 mRNA Covid-19 vaccine. N Engl J Med.

[4] European Medicines Agency (2021). Comirnaty (COVID-19 mRNA VACCINE) risk management plan. Risk Management Plan.

[5] Adler Y, Charron P, Imazio M (2015). 2015 ESC Guidelines for the diagnosis and management of pericardial diseases. Kardiol Pol.

[6] Imazio M, Brucato A, Lazaros G (2020). Anti-inflammatory therapies for pericardial diseases in the COVID-19 pandemic: safety and potentiality. J Cardiovasc Med (Hagerstown).

[7] Cremer PC, Kumar A, Kontzias A, Tan CD, Rodriguez ER, Imazio M, Klein AL (2016). Complicated pericarditis: understanding risk factors and pathophysiology to inform imaging and treatment. J Am Coll Cardiol.

[8] Lazaros G, Anastassopoulou C, Hatziantoniou S (2021). A case series of acute pericarditis following COVID-19 vaccination in the context of recent reports from Europe and the United States. Vaccine.

[9] Madhi SA, Baillie V, Cutland CL (2021). Efficacy of the ChAdOx1 nCoV-19 Covid-19 vaccine against the B.1.351 variant. N Engl J Med.

[10] Sadoff J, Gray G, Vandebosch A (2021). Safety and efficacy of single-dose Ad26.COV2.S vaccine against Covid-19. N Engl J Med.

[11] Mane VP, Toietta G, McCormack WM (2006). Modulation of TNFalpha, a determinant of acute toxicity associated with systemic delivery of first-generation and helper-dependent adenoviral vectors. Gene Ther.

[12] Lazaros G, Klein AL, Hatziantoniou S, Tsioufis C, Tsakris A, Anastassopoulou C (2021). The novel platform of mRNA COVID-19 vaccines and myocarditis: clues into the potential underlying mechanism. Vaccine.

